# Double Fetation

**Published:** 1878-05

**Authors:** 


Double Fetation.—On the evening of May 23, 1869, I was requested to visit Mrs. N., aged nineteen years, a primipara. I was informed by her mother that "they supposed Mrs. N. to be pregnant since the latter part of January; that she commenced wasting in the morning, and was now flooding fearfully." I found the patient propped up in bed, and well nigh exsanguine. I immediately removed the pillows from under her head and shoulders, and ordered the foot of the bed raised some ten or twelve inches. This done I made a digital examination, and found the mouth of the womb dilated to the size of a silver half dollar, and readily detected a central placental presentation. Her condition was such that I deemed it advisable to use the tampon, which was thoroughly applied, entirely arresting further drainage. This accomplished I took a seat to await results, remaining by her bedside until the next morning; and as there was neither hemorrhage nor pain, I enjoined perfect quiet, and left with instructions to be summoned if necessary. I did not see her again until six o'clock in the evening, when I found her in active labor pains, and soon tampon, placenta and fetus were expelled. I also removed from the vagina a well compressed coagulum, the size of a hen's egg, being the entire amount of hemorrhage occurring since the application of the tampon. From this on, my patient made a rapid recovery, and the case faded from my mind as one of placenta prævia with fortunate termination.

On the thirtieth day of October following, I was again summoned to visit Mrs. N. This time I found her in active labor, and within two hours from the time of my arrival she was delivered of a live female child, weighing nine pounds and eight ounces. The child is now a bright little school-girl of nine summers, her birth taking place just five months and six days after the unfortunate expulsion of her twin sister.

Placenta prævia, with excessive flooding, use of tampon, expulsion of the dead and retention of the living twin to full term, are points of professional interest.—*American Practitioner.*

